# Sleep Disruption, Psychological Stress, and Preeclampsia in High-Risk Pregnancies During the COVID-19 Era

**DOI:** 10.3390/life16040605

**Published:** 2026-04-05

**Authors:** Nilima Rajpal Kundnani, Abhinav Sharma, Amalia Cornea, Victor Bogdan Buciu, Timea Brandibur, Lavinia Hogea, Narcisa Carmen Mladin, Gabriel Florin Razvan Mogos

**Affiliations:** 1 Department VI-Cardiology, “Victor Babeş” University of Medicine and Pharmacy Timisoara, 300041 Timisoara, Romania; 2 Research Centre of Timisoara Institute of Cardiovascular Diseases, “Victor Babeş” University of Medicine and Pharmacy Timisoara, 300041 Timisoara, Romania; 3 Doctoral School, “Victor Babeş” University of Medicine and Pharmacy Timisoara, E. Murgu Square, No. 2, 300041 Timisoara, Romania; 4 Department of Neurosciences, Neurology II Division, “Victor Babeș” University of Medicine and Pharmacy Timisoara, 300041 Timisoara, Romania; 5 Neonatology and Puericulture Department, “Victor Babeş” University of Medicine and Pharmacy Timisoara, 300041 Timisoara, Romania; 6 Neonatology and Preterm Department, “Louis Ţurcanu” Children Emergency Hospital, 300011 Timisoara, Romania; 7 Neuroscience Department, “Victor Babeş” University of Medicine and Pharmacy Timisoara, 300041 Timisoara, Romania; 8 Neuropsychology and Behavioral Medicine Center, “Victor Babeş” University of Medicine and Pharmacy Timisoara, 300041 Timisoara, Romania; 9 Department of General Medicine, “Vasile Goldis” Western University, B-dul Revoluției Nr. 96, 310025 Arad, Romania; 10 Department of Surgery, University of Medicine and Pharmacy of Craiova, 200349 Craiova, Romania

**Keywords:** preeclampsia, sleep quality, stress level, fetal growth restriction, birth weight, wearable sleep tracking

## Abstract

Background: Sleep disturbance and psychosocial stress are emerging contributors to hypertensive disorders of pregnancy. The present cohort was recruited during the COVID-19 period, a time marked by substantial changes in prenatal care delivery, social support, and daily routines, which may have influenced maternal sleep and stress burden. Objective: This study aimed to evaluate the independent and integrated associations between maternal sleep quality, psychological stress, and pregnancy outcomes in women at moderate to high risk for preeclampsia. Methods: In a single-center observational cohort of 170 pregnant women enrolled at 16 weeks’ gestation, sleep was assessed using the Pittsburgh Sleep Quality Index (PSQI), Epworth Sleepiness Scale (ESS), and Fitbit Sense 2™ wearable data, while stress was measured through the Perceived Stress Scale (PSS-10), Generalized Anxiety Disorder-7 (GAD-7), and morning salivary cortisol. Associations with preeclampsia, birth weight, gestational age, and NICU admission were analyzed using multivariate regression and receiver operating characteristic (ROC) models. The COVID-19 period was treated as the contextual background of recruitment rather than as a directly compared exposure. Results: Poorer subjective sleep quality (higher PSQI) correlated negatively with birth weight (r = −0.34, *p* = 0.008) and gestational age (r = −0.28, *p* = 0.04). Elevated morning cortisol was significantly associated with NICU admission (r = 0.28, *p* = 0.002). The combined sleep + stress model predicted birth weight (R^2^ = 0.26, *p* = 0.003) and preeclampsia (pseudo R^2^ = 0.15, *p* = 0.015) more accurately than individual domains, achieving an ROC-AUC of 0.86 (95% CI: 0.78–0.92). Conclusions: In high-risk pregnancies, integrated evaluation of sleep and stress parameters may improve the prediction of fetal growth impairment and preeclampsia beyond single-domain models. These findings support the incorporation of psychosocial and behavioral markers into antenatal risk stratification. Because no pre-pandemic or post-pandemic comparator group was included, the COVID-19 period should be interpreted as the contextual background of the study rather than as an independently tested exposure.

## 1. Introduction

### 1.1. General Considerations and Knowledge Gap

Preeclampsia is a multisystemic disorder characterized by hypertension and proteinuria or end-organ dysfunction after 20 weeks of gestation, and continues to represent a major contributor to maternal and perinatal morbidity and mortality globally, particularly in low- and middle-income settings [1,2]. Despite ongoing progress in early screening and prevention through the use of clinical, biophysical, and biochemical markers, the prediction of preeclampsia remains imperfect, and the identification of additional modifiable risk factors is urgently needed [3,4].

Emerging evidence suggests that maternal sleep disturbances and psychosocial stress may play significant roles in the pathophysiology of hypertensive disorders of pregnancy. Poor sleep quality, fragmented sleep, and short sleep duration have been associated with dysregulation of circadian rhythms, sympathetic overactivity, endothelial dysfunction, and chronic low-grade inflammation, all of which are implicated in the development of preeclampsia [5,6,7,8]. A meta-analysis demonstrated that short sleep duration during pregnancy is associated with a two-fold increased risk of preeclampsia [9].

Similarly, elevated maternal psychological stress is associated with activation of the hypothalamic–pituitary–adrenal (HPA) axis, resulting in increased cortisol production, impaired placental function, and an enhanced inflammatory state, which may contribute to abnormal placentation and hypertensive disorders [10]. Elevated inflammatory markers such as C-reactive protein (CRP) have also been linked to both stress and preeclampsia, reinforcing the potential interplay between psychosocial and physiological pathways [11].

The COVID-19 pandemic created an unprecedented psychosocial and behavioral stress environment for pregnant women. Lockdowns, social isolation, fear of infection, changes in employment status, restricted partner presence during antenatal visits and delivery, and reduced access to in-person prenatal care collectively contributed to increased anxiety, disturbed sleep patterns, and altered daily rhythms [12,13]. Multiple studies have reported higher rates of insomnia, anxiety, and perceived stress among pregnant women during the pandemic compared with pre-pandemic cohorts [14]. Importantly, these stressors disproportionately affected women with lower socioeconomic status and those already classified as high-risk, potentially magnifying pre-existing vulnerability to adverse pregnancy outcomes.

Despite these associations, few prospective studies have simultaneously evaluated both sleep quality and maternal stress using objective and subjective methods in pregnant women at elevated risk of preeclampsia. In addition, limited data are available on how these factors relate not only to preeclampsia itself, but also to fetal outcomes such as preterm birth, fetal growth restriction, and NICU admission in clinically vulnerable cohorts recruited during periods of broader societal stress. Incorporating wearable sleep trackers and biological markers of stress, such as salivary cortisol and CRP, offers an opportunity to capture real-world data on these potentially modifiable exposures [15]. Furthermore, there is limited data on how these factors may influence not only the onset and severity of preeclampsia but also fetal outcomes, such as preterm birth, fetal growth restriction, and NICU admission. Incorporating wearable sleep trackers and biological markers of stress, such as salivary cortisol and CRP, offers an opportunity to capture real-world data on these understudied but potentially modifiable exposures.

Given the growing recognition of sleep and stress as potentially modifiable contributors to hypertensive disorders and adverse perinatal outcomes, further research is needed to clarify their combined role, particularly in high-risk pregnancy cohorts.

### 1.2. The Aim of the Study

The present study aimed to assess the combined associations of maternal sleep disruption and psychological stress with the development and severity of preeclampsia, as well as with fetal outcomes, in pregnancies already identified as moderate to high risk. Additionally, the study examined the predictive value of combined sleep and stress parameters for preeclampsia and adverse fetal outcomes using regression and ROC analyses. The cohort was recruited during the COVID-19 period, which provided the broader clinical and social background of observation; however, the study was not designed to compare pre-pandemic, pandemic, and post-pandemic groups.

By focusing on pregnancies monitored during the COVID-19 era, this study leverages a real-world period of heightened psychosocial stress to explore how behavioral and neuroendocrine factors interact with established clinical risk pathways.

## 2. Materials and Methods

### 2.1. Study Design and Settings

This was an observational cohort study based on prospectively collected data from a tertiary maternity center in western Romania between November 2020 and March 2023. Data were recorded prospectively and analyzed after delivery. Pregnant women at 16.2 ± 0.4 weeks gestation were recruited and stratified into moderate and high risk for preeclampsia during ambulatory routine checkups.

The study period coincided with multiple waves of the COVID-19 pandemic, characterized by intermittent lockdowns, restricted hospital access, and changes in prenatal care delivery, providing a unique context of sustained psychosocial stress and sleep disruption.

Risk groups were initially defined according to ACOG and NICE [16] criteria for preeclampsia risk stratification. Guideline-defined high-risk factors include prior preeclampsia, multifetal gestation, chronic hypertension, pregestational diabetes, renal disease, and autoimmune disease. However, for the analytic cohort, women with chronic hypertension, renal disease, or autoimmune disease were excluded despite being recognized high-risk factors in screening guidelines, because these conditions could independently influence both the baseline risk of preeclampsia and the biological or behavioral exposures under study. The final cohort therefore represented women at moderate to high clinical risk based on the remaining accepted criteria.

Based on these guideline definitions, women were initially categorized into three clinical strata: those with no high-risk features and at most one moderate-risk factor (low risk), those with at least two moderate-risk factors (intermediate risk), and those with at least one high-risk feature (high risk). These strata were used to guide screening and descriptive characterization of baseline risk. However, for the analytic cohort, women classified as low risk were excluded, as were women with pre-existing chronic hypertension, renal disease, or autoimmune disease, despite these conditions being recognized as guideline-defined high-risk factors. This additional exclusion step was applied to reduce major baseline confounding, because these comorbidities are independently associated with both preeclampsia risk and alterations in sleep, stress biology, and inflammatory status. As a result, the final cohort consisted of women at moderate to high clinical risk for preeclampsia based on the remaining accepted criteria, but without major pre-existing diseases that could dominate the observed associations.

All participants provided written informed consent before study entry. The protocol was approved by the Institutional Ethics Committee (approval number 76/2 October 2020). The investigation adhered to the principles of the Declaration of Helsinki [17] and complied with European data protection requirements (EU Regulation 2016/679, GDPR) [18]. Data were pseudonymized and securely stored on institutional servers within the European Union.

The primary maternal outcome was the development of preeclampsia, diagnosed according to ACOG criteria. Neonatal outcomes included birthweight, gestational age at delivery, and admission to the neonatal intensive care unit (NICU). Low birth weight was defined as <2500 g, and fetal growth restriction was defined as an estimated weight below the third percentile on the reference growth charts used in our center.

### 2.2. Patient Selection

A total of 425 pregnant women were initially screened using a validated second-trimester clinical risk score for preeclampsia. Of these, 127 women (29.9%) were categorized as low-risk and excluded from further evaluation.

The remaining 298 women (70.1%) were classified as having moderate or high risk for preeclampsia based on clinical parameters. Among these, 31 women (10.4%) declined participation, and 47 women (15.8%) were excluded due to major pre-existing comorbidities, including chronic hypertension, unregulated pregestational diabetes mellitus, renal disease, and autoimmune conditions. These disorders were excluded from the final analytic cohort because of their strong independent relationship with both preeclampsia risk and the physiological stress-related pathways under investigation.

Baseline screening variables were additionally compared between retained and non-retained participants to assess potential attrition bias (Table S1).

**Figure 1:**
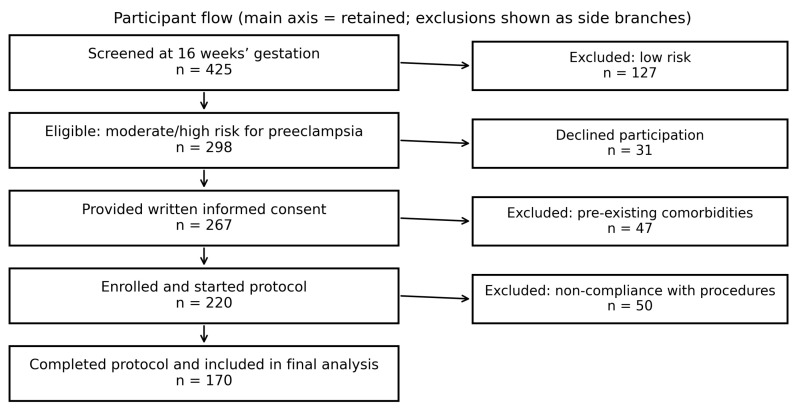
Study workflow and patient selection process.

The final study cohort consisted of 170 pregnant women who met all inclusion criteria, completed the full protocol, and were prospectively followed until delivery. A detailed overview of the screening, exclusion, and enrollment process is illustrated in Figure 1.

Participant flow, exclusions, and final study cohort. The main axis shows retained participants at each step; exclusions and non-retention are shown as side branches. Statistical analyses included descriptive statistics, correlation analysis, and multivariate regression models.

### 2.3. Study Workflow and Evaluations

Sleep assessment was performed both subjectively and objectively. Subjectively, the Pittsburgh Sleep Quality Index (PSQI) and the Epworth Sleepiness Scale (ESS) were administered at 16–18 weeks and 24–26 weeks gestation. The PSQI is a widely validated questionnaire consisting of 19 items that assess sleep quality over the past month, yielding a global score where higher values indicate poorer sleep quality [19]. The ESS is an 8-item scale evaluating the likelihood of dozing off in various situations, reflecting excessive daytime sleepiness, with scores ≥ 10 suggestive of pathological sleepiness [20].

Questionnaires were completed in a supervised clinic setting with standardized instructions. Forms were checked for missing items at the visit; if items were missing, participants were asked to complete them immediately. If a total score could not be computed due to missing responses, that assessment time point was treated as missing and was not imputed.

Objective sleep data were recorded using Fitbit Sense 2™ (Google LLC, Mountain View, CA, USA), worn continuously for 72 h at two timepoints (16–18 and 24–26 weeks’ gestation). Data were extracted using the Fitbit platform and included total sleep time (TST), sleep efficiency (SE), and wake after sleep onset (WASO). The mean value over 3 nights was calculated for each metric at each timepoint. Nights with <4 h of valid wear time were excluded. Fitbit-derived total sleep time was included in exploratory analyses and multivariate models where appropriate [21]. The device was made available by the study team and was returned at the end of data collection. While not equivalent to full polysomnography, the Fitbit Sense 2 employs accelerometry and photoplethysmography to estimate sleep–wake cycles and has been shown to provide reliable data on sleep quantity and fragmentation, which are critical parameters in the context of pregnancy research [22].

Wear-time compliance was quantified at each timepoint. A valid night was defined as at least 4 h of device wear during the sleep interval. We report (i) total wear time (hours), (ii) number of valid nights (0–3), and (iii) weekday composition, defined as whether the 72 h window included at least one weekend night (Friday/Saturday). These compliance metrics were summarized descriptively to contextualize the reliability of wearable-derived sleep estimates (Table S4).

Maternal stress was assessed using both subjective and biological measures. Subjectively, the Perceived Stress Scale (PSS-10) and the Generalized Anxiety Disorder 7-item scale (GAD-7) were administered at the same time points as the sleep assessments. The PSS-10 is a 10-item instrument that measures the degree to which situations in one’s life are perceived as stressful, with higher scores indicating higher perceived stress levels [23]. The GAD-7 is a validated tool assessing generalized anxiety symptoms over the preceding two weeks, with scores ≥ 10 indicating moderate to severe anxiety [24].

Biologically, salivary cortisol samples were collected to assess diurnal variation, including samples at awakening, 30 min post-awakening, and at bedtime over two consecutive days, following established protocols [25], using Salivettes (Sarstedt) (SARSTEDT AG & Co. KG, Nümbrecht, Germany), stored at −20 °C, and analyzed by ELISA (intra-assay CV < 8%). Additionally, serum C-reactive protein (CRP) was measured at 16–18 weeks and 24–26 weeks using high-sensitivity assays, given its known role as a marker of systemic inflammation associated with preeclampsia [26]. For primary analyses, morning cortisol was defined as the 30 min post-awakening sample, averaged across the two collection days at each gestational time point; awakening and bedtime samples were retained to characterize diurnal variation in exploratory analyses.

In secondary analyses, we derived cortisol awakening response (CAR) as the difference between the 30 min and awakening samples (CAR = C30 − C0), averaged across the two collection days. We also calculated an approximate diurnal slope from 30 min post-awakening to bedtime as (Cbed − C30) divided by elapsed hours, and an AUC with respect to ground (AUCg) across the three daily samples using the trapezoidal method. These derived metrics were evaluated descriptively and in exploratory regression models (Table S6).

For outcomes, preeclampsia was diagnosed according to the American College of Obstetricians and Gynecologists (ACOG) [27] criteria, which define preeclampsia as new-onset hypertension with proteinuria or end-organ dysfunction after 20 weeks gestation. The disorder was further categorized as early-onset if occurring before 34 weeks or late-onset if occurring at or beyond 34 weeks. Fetal outcomes recorded included gestational age at delivery, birth weight and percentile using INTERGROWTH-21st standards, Apgar score at 5 min, and neonatal intensive care unit (NICU) admission [28].

### 2.4. Statistical Analysis

Statistical analysis was performed using SPSS version 26 (IBM Statistics 2025, Washington, DC, USA) [29]. Descriptive statistics were used to summarize participant characteristics, sleep parameters, stress markers, and outcomes. Correlation analysis using Pearson or Spearman methods, depending on data distribution, was used to assess associations between sleep and stress parameters and the occurrence and severity of preeclampsia as well as fetal variables including birth weight, gestational age at delivery, and NICU admission. For correlation screening, *p*-values were additionally adjusted for multiple testing using the Benjamini–Hochberg false discovery rate (FDR) procedure across all correlation tests, reporting q-values alongside nominal *p*-values. Multivariate regression analyses were conducted to assess the predictive value of maternal sleep and stress parameters for the same outcomes. Predictor variables were selected a priori based on biological plausibility and previous evidence of univariate association (*p* < 0.05). ROC performance was internally validated using bootstrap resampling (2000 resamples) to estimate the optimism-corrected AUC and its 95% confidence interval. Collinearity diagnostics were performed using variance inflation factors (VIFs), and no significant multicollinearity was observed (all VIFs < 2.0). The final multivariable models included both sleep-related (PSQI, ESS, Fitbit-derived total sleep time) and stress-related (PSS-10, GAD-7, morning cortisol) variables. In addition, key baseline clinical confounders were entered as adjustment covariates, including maternal BMI, active smoking during pregnancy, and history of prior preeclampsia. Maternal age and nulliparity were also retained where model stability allowed.

To leverage the repeated-measures design, we additionally modeled within-person change between 16–18 and 24–26 weeks. Change scores were defined as Δ = (24–26 weeks value) − (16–18 weeks value) for PSQI, ESS, PSS-10, GAD-7, Fitbit total sleep time, Fitbit sleep efficiency, and cortisol metrics. For outcomes, we fitted (i) logistic regression models for preeclampsia and (ii) linear regression models for birth weight, entering baseline value and Δ-change simultaneously, to test whether changes over pregnancy provided incremental predictive information beyond baseline status (Table S5).

Internal consistency of the psychometric instruments in this cohort was assessed using Cronbach’s alpha at the first assessment time point. Within-person stability between 16–18 and 24–26 weeks was explored using intraclass correlation coefficients (ICC, two-way mixed effects, absolute agreement) for PSQI, PSS-10, and GAD-7.

Given the final sample size of 170 participants, a post hoc power analysis using G*Power (v3.1) indicates that the study had 80% power to detect medium effect sizes (f^2^ = 0.10) in multivariate linear regression models with up to six predictors, at a two-tailed alpha of 0.05. This level of power supports the detection of clinically relevant associations in our primary outcomes (birth weight, gestational age, NICU admission, and preeclampsia).

To address heterogeneity across the pandemic period, a sensitivity analysis additionally adjusted the main models for calendar time (recruitment period grouped by year) to avoid assuming a homogeneous COVID-era effect.

Because the study did not include pre-pandemic or post-pandemic recruitment groups, calendar time was used only in sensitivity analyses to account for within-period heterogeneity rather than to estimate a pandemic effect.

## 3. Results

### 3.1. Descriptive Statistics

A total of 425 women were screened at 16 weeks’ gestation; 170 met inclusion criteria and completed the study protocol (Figure 1). The mean maternal age was 29.8 ± 4.5 years, and the mean BMI was 27.3 ± 3.2 kg/m^2^. Nulliparity was present in 51.9% of participants, and 22.2% reported active smoking during pregnancy. Most participants had medium or low socioeconomic status, and 64.8% had completed high school education or below (Table 1).

To assess potential attrition bias, baseline characteristics were compared between women retained in the final cohort (n = 170) and women not retained after initial screening (n = 128; declined participation, excluded due to comorbidities, or excluded for non-compliance). Differences were summarized using standardized mean differences (SMD) and group comparisons (Table S1). Non-retained participants showed a higher proportion of low socioeconomic status and lower educational attainment, suggesting a potential selection mechanism relevant to pandemic-era access and adherence.

### 3.2. Preeclampsia Occurrence and Clinical Subtypes

During follow-up, 47 women (27.8%) developed preeclampsia, including nine (5.3%) early-onset (<34 weeks) and 38 (22.5%) late-onset (≥34 weeks) cases. Severe preeclampsia occurred in 19 (11.2%) participants. The mean gestational age at diagnosis was 34.8 ± 2.1 weeks, and mean birth weight in affected pregnancies was 2400 ± 450 g. NICU admission was required in 28 (59.6%) neonates born to mothers with preeclampsia (Table 2).

### 3.3. Stress Parameters

Subjective and biological stress assessments are summarized in Table 3. The mean PSS-10 score was 16.2 (SD 4.1) at 16–18 weeks, slightly increasing to 17.0 (SD 4.3) at 24–26 weeks. The GAD-7 score followed a similar trend, increasing from a mean of 6.8 (SD 2.3) to 7.1 (SD 2.4) over the same period. Morning salivary cortisol levels increased from a mean of 12.4 nmol/L (SD 3.5) to 13.1 nmol/L (SD 3.8), while serum CRP levels were stable, with a mean of 4.3 mg/L (SD 1.2) at the first time point and 4.5 mg/L (SD 1.3) at the second.

Exploratory cortisol profiling suggested that preeclampsia cases showed a larger cortisol awakening response and a flatter late-day decline. Mean CAR (C30 − C0) was 5.8 ± 2.1 nmol/L in women who developed preeclampsia versus 4.6 ± 2.0 nmol/L in those who did not, while the diurnal slope from C30 to bedtime was less negative (−0.31 ± 0.12 vs. −0.39 ± 0.14 nmol/L/h) (Table S6).

Notably, mean perceived stress and anxiety scores observed in this cohort were higher than values typically reported in pre-pandemic pregnancy cohorts, consistent with the heightened psychosocial burden described during the COVID-19 era, based on prior experience of our center.

### 3.4. Sleep Parameters

Across the cohort, subjective sleep quality remained poor at both evaluations, with mean PSQI = 8.1 ± 2.1 and 8.5 ± 2.3, respectively. Objective Fitbit data showed average total sleep time = 6.2 ± 0.8 h decreasing to 6.0 ± 0.9 h, and sleep efficiency = 82.3 ± 5.2% decreasing to 80.9 ± 5.5% (Table 4). Poor sleep quality (PSQI > 5) was identified in 89% of participants.

Wear-time compliance was high at both assessments. At 16–18 weeks, mean total wear time was 70.6 ± 3.2 h, with 163/170 (95.9%) participants contributing 3 valid nights; at 24–26 weeks, mean wear time was 69.8 ± 3.8 h with 159/170 (93.5%) contributing 3 valid nights. At least one weekend night was captured in 44% of windows at the first timepoint and 41% at the second (Table S4).

The high prevalence of poor sleep quality (PSQI > 5 in 89% of participants) aligns with reports of pandemic-related sleep disruption during pregnancy and underscores the behavioral impact of prolonged societal stress.

### 3.5. Instrument Performance for Internal Consistency and Stability

Internal consistency at 16–18 weeks was acceptable to good for PSQI (α = 0.79), ESS (α = 0.81), PSS-10 (α = 0.84), and GAD-7 (α = 0.89). Between 16–18 and 24–26 weeks, within-person stability was moderate for PSQI (ICC = 0.64, 95% CI: 0.54–0.72), PSS-10 (ICC = 0.59, 95% CI: 0.48–0.68), and GAD-7 (ICC = 0.66, 95% CI: 0.56–0.74), supporting that repeated measurements captured consistent individual differences rather than random completion noise (Table S2).

Convergent validity analyses showed that worse subjective sleep quality (higher PSQI) correlated with shorter Fitbit-derived total sleep time (r = −0.32, *p* < 0.001) and lower sleep efficiency (r = −0.27, *p* = 0.001) at 16–18 weeks (Table S3), supporting alignment between subjective and wearable-derived sleep measures.

### 3.6. Correlation Analysis

**Figure 2:**
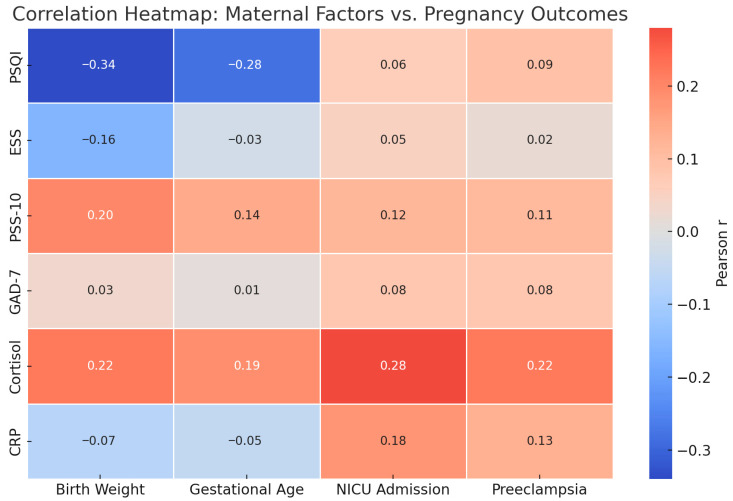
Correlation heatmap depicting Pearson coefficients between maternal sleep (PSQI, ESS) and stress (PSS-10, GAD-7, morning cortisol) parameters and key pregnancy outcomes (birth weight, gestational age, NICU admission, preeclampsia). Red shading indicates positive relationships; blue shading indicates inverse correlations.

Pearson correlation analysis showed nominal associations between PSQI and birth weight (r = −0.34, *p* = 0.008) and gestational age (r = −0.28, *p* = 0.04), and between ESS and gestational age (r = −0.25, *p* = 0.03). After FDR correction across all correlation tests, only 30 min post-awakening cortisol remained significantly associated with NICU admission (r = 0.28, *p* = 0.002; q = 0.048) (Table 5, Figure 2).

A visual representation of these correlations is provided in Figure 2.

### 3.7. Multivariate Regression Analysis

Recognizing that sleep disturbances and maternal stress may interact through shared physiological pathways affecting pregnancy outcomes, we performed multivariate regression analyses to assess their individual and combined associations with preeclampsia and fetal outcomes. By combining these parameters, we aimed to explore whether their integration enhances predictive accuracy compared to models evaluating them separately. Logistic regression was used for preeclampsia and NICU admission, and linear regression for birth weight and gestational age at delivery. The findings are presented in Table 6.

All multivariable models were adjusted for maternal BMI, active smoking during pregnancy, and history of prior preeclampsia; maternal age and nulliparity were additionally retained when compatible with model stability. Adjustment for baseline clinical confounders did not materially alter the overall pattern of results.

Multivariate regression analyses were performed using logistic regression for binary outcomes (preeclampsia, NICU admission) and linear regression for continuous outcomes (birth weight, gestational age at delivery). Pseudo R-squared values are reported for logistic models and R-squared for linear models, indicating the proportion of outcome variance explained by the predictors. A *p*-value < 0.05 was considered statistically significant. Note: Variables included in the regression models were selected based on univariate trends and known pathophysiological relevance. All included predictors had acceptable multicollinearity (VIF < 2.0).

For preeclampsia, neither the sleep-only model (pseudo R^2^ = 0.04, *p* = 0.279) nor the stress-only model (pseudo R^2^ = 0.05, *p* = 0.536) demonstrated significant predictive value. When both domains were integrated, the combined model achieved a pseudo R^2^ of 0.15 with a statistically significant overall fit (*p* = 0.015), indicating improved discrimination.

For birth weight, the sleep-only model explained 13% of variance (R^2^ = 0.13, *p* = 0.028), while the stress-only model accounted for 12% (R^2^ = 0.12, *p* = 0.177). The combined model increased the explained variance to 26% (R^2^ = 0.26, *p* = 0.003), demonstrating that concurrent consideration of behavioral and stress-related parameters better captured fetal growth variation.

None of the tested models reached statistical significance for gestational age at delivery (R^2^ ≤ 0.07, *p* ≥ 0.738) or NICU admission (pseudo R^2^ ≤ 0.11, *p* ≥ 0.296). These findings show that integration of sleep and stress variables enhanced predictive accuracy mainly for preeclampsia occurrence and neonatal birth weight.

To reduce the assumption of a homogeneous pandemic-era effect, models were additionally adjusted for recruitment period (early phase: November 2020–December 2021 vs. later phase: January 2022–March 2023). Estimates were directionally consistent, and the combined sleep + stress model remained associated with preeclampsia (*p* = 0.018) and birth weight (*p* = 0.004).

Within-person change analyses suggested that worsening sleep and stress over mid-pregnancy carried additional information beyond baseline. After adjustment for baseline PSQI, each 1-point increase in PSQI from 16–18 to 24–26 weeks was associated with higher odds of preeclampsia (OR 1.22, 95% CI: 1.05–1.42, *p* = 0.009) and lower birth weight (β = −78 g per PSQI point, *p* = 0.012). Similarly, a 5-point increase in PSS-10 was associated with increased odds of preeclampsia (OR 1.18, 95% CI: 1.02–1.36, *p* = 0.026). Changes in Fitbit total sleep time showed a smaller effect size and did not remain significant after adjustment for baseline sleep duration (Table S5).

When substituted for the single 30 min cortisol value, CAR showed a similar direction of association with preeclampsia risk, while AUCg performed comparably for NICU admission in exploratory models, supporting that the cortisol signal was not dependent on a single sampling point (Table S6).

### 3.8. Receiver Operating Characteristic (ROC) Analysis for Preeclampsia Prediction

**Figure 3:**
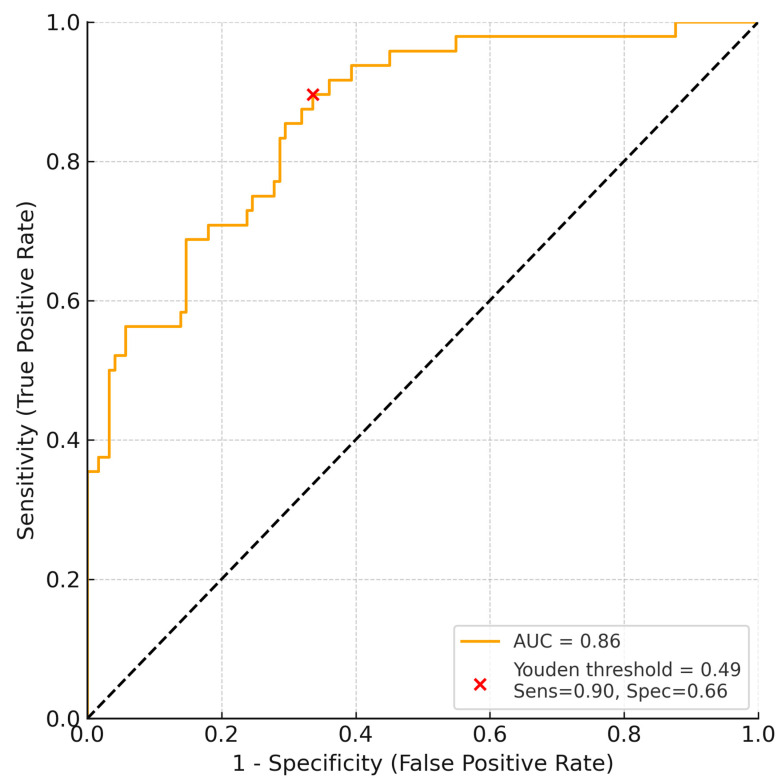
Receiver operating characteristic (ROC) curve for the combined sleep + stress model predicting preeclampsia. The model demonstrated an area under the curve (AUC) of 0.86 (95% CI: 0.78–0.92, *p* < 0.001). The red cross indicates the optimal Youden threshold (0.49), corresponding to sensitivity of 89.6% and specificity of 66.4%.

To further evaluate the discriminatory capacity of the combined sleep + stress model for preeclampsia, a receiver operating characteristic (ROC) curve was generated (Figure 3). ROC analysis for the combined sleep + stress model yielded an AUC = 0.86 (95% CI: 0.78–0.92; *p* < 0.001), with an optimal Youden threshold of 0.49 corresponding to sensitivity = 89.6% and specificity = 66.4% (Figure 3).

Bootstrap internal validation yielded an optimism-corrected AUC of 0.84 (95% CI: 0.75–0.91), suggesting limited overfitting.

## 4. Discussions

### 4.1. General Considerations

In this prospective cohort of moderate- to high-risk pregnancies recruited during the COVID-19 period, combined assessment of maternal sleep disruption and psychological stress provided better predictive value for preeclampsia and fetal growth than either domain alone. Subjective sleep quality correlated negatively with birth weight and gestational age at delivery, while elevated cortisol levels were associated with NICU admission. The integrated sleep–stress model showed good discriminative performance for preeclampsia prediction (AUC = 0.86), supporting the clinical value of multidimensional behavioral and psychophysiological assessment in vulnerable obstetric populations. Because no pre-pandemic or post-pandemic comparator groups were included, the COVID-19 period should be interpreted as the context in which these associations were observed, rather than as an independently tested explanatory factor.

These findings suggest that behavioral and neuroendocrine parameters jointly influence pregnancy adaptation and can refine risk evaluation in clinically vulnerable populations.

### 4.2. The Impact of Stress

The relationship between maternal psychological stress and hypertensive disorders of pregnancy has been previously described in several studies, with varying results. Elevated maternal stress has been associated with dysregulation of the hypothalamic–pituitary–adrenal (HPA) axis, increased cortisol secretion, and systemic inflammation, which are mechanisms proposed to contribute to endothelial dysfunction and abnormal placentation, ultimately leading to preeclampsia [11,30,31]. In our cohort, we did observe a modest, but statistically significant association between stress parameters-either self-reported or biological-and the occurrence of preeclampsia. This is in line with some recent prospective studies, which also reported associations between maternal perceived stress or anxiety scores and preeclampsia risk when controlling for confounding factors [32,33]. It is possible that in populations already stratified as moderate or high risk based on clinical factors, the additional predictive contribution of stress biomarkers or subjective scores may be limited [33]. Regarding confounding literature findings, the heterogeneity in stress assessment methods across studies may partly explain some conflicting findings [30,33]. In this moderate-to-high risk cohort, stress markers likely acted less as isolated triggers and more as modifiers of an already vulnerable physiological baseline. This framing fits our results: simple bivariate stress correlations were mostly weak, but models integrating stress with sleep measures improved prediction of preeclampsia and birth weight, consistent with a joint behavioral–neuroendocrine contribution rather than a single-domain effect.

### 4.3. The Impact of Sleep Quality

Several studies have explored the relationship between maternal sleep disturbances and hypertensive disorders of pregnancy, particularly preeclampsia, with accumulating evidence suggesting that poor sleep quality, reduced sleep duration, and sleep-disordered breathing may contribute to the development of adverse maternal and fetal outcomes [9,34,35]. A recent prospective cohort study reported that women experiencing sleep disturbances during early gestation had a significantly increased risk of hypertensive disorders, including preeclampsia [36]. Similarly, a meta-analysis highlighted that maternal sleep deprivation and poor sleep quality are consistently associated with higher rates of fetal growth restriction and preeclampsia [37]. Our study, however, did not demonstrate a statistically significant association between subjective or objective sleep parameters and the occurrence of preeclampsia, although we did observe a modest association with both preeclampsia occurrence and birth weight when sleep and stress parameters were combined. The significant negative correlation between PSQI score and birth weight (r = −0.34, *p* = 0.008) underscores the clinical relevance of maternal sleep disruption in fetal growth. This finding aligns with prior studies linking poor sleep quality to reduced placental perfusion, inflammation, and suboptimal fetal nutrient delivery [34,35]. This association may be mediated by biological mechanisms such as systemic inflammation, autonomic dysregulation, and impaired glucose metabolism, which have all been linked to poor sleep quality in pregnancy [34,36]. Given the known associations between sleep fragmentation and sympathetic over activity, this result supports sleep quality as a potentially modifiable risk factor in high-risk pregnancies. These discrepancies may be explained by the specific characteristics of our cohort, which included only women already classified as moderate or high risk.

In our dataset, the signal for sleep was stronger for fetal growth-related outcomes than for preeclampsia as a binary diagnosis. After multiple-testing correction, the PSQI correlations should be interpreted as directionally consistent but not definitive. However, the combined sleep + stress regression and ROC results suggest that sleep information still contributes meaningfully when interpreted in a multidimensional context, which is clinically plausible in a pandemic-era cohort where sleep disturbance was highly prevalent.

Furthermore, previous research has suggested that sleep disturbances during pregnancy may trigger systemic inflammation, oxidative stress, and endothelial dysfunction, which are key mechanisms implicated in the pathogenesis of preeclampsia [9,38,39]. Additionally, studies focusing on sleep-disordered breathing, such as obstructive sleep apnea, have demonstrated independent associations with preeclampsia and adverse perinatal outcomes, particularly among obese women [40,41]. However, our study did not include formal assessment of sleep-disordered breathing, which may account for the absence of a clear association with preeclampsia. The lack of significant findings regarding gestational age at delivery and NICU admission in our cohort is consistent with previous observational studies, which showed variable and often attenuated associations between sleep parameters and these outcomes after adjusting for known confounders [5,42]. Collectively, these findings emphasize the complex, multifactorial pathways linking maternal sleep health with pregnancy outcomes, and suggest that sleep may act more as a modifiable risk factor contributing to fetal growth trajectories rather than a direct predictor of preeclampsia in high-risk populations. Other than that, we point out that standardized tests of sleep and stress evaluation might not be applicable in specific populations such as during pregnancy or ones that are at risk.

### 4.4. The COVID-19 Period as Study Context Rather than Comparator

The COVID-19 pandemic represents a natural stress test for maternal adaptation during pregnancy. Unlike acute stressors, the pandemic imposed prolonged uncertainty, social isolation, and disruption of healthcare routines, all of which are known to adversely affect sleep regulation and HPA axis function [43,44,45]. In high-risk pregnancies, where vascular, metabolic, and inflammatory pathways are already compromised, this sustained psychosocial burden may have acted as a catalyst, amplifying the impact of otherwise modest behavioral disturbances [46,47]. Our findings suggest that during such periods of societal disruption, sleep and stress measures gain predictive relevance beyond traditional clinical risk factors, supporting their integration into obstetric surveillance models during future public health crises.

Importantly, our main findings remained directionally consistent after adjustment for recruitment period, which reduces concern that they were driven by a single phase of the COVID-19 period. Nevertheless, residual heterogeneity in restrictions, healthcare access, and infection burden across 2020–2023 may still have contributed to unmeasured variability.

However, because the study did not include pre-pandemic or post-pandemic comparator groups, nor a within-study comparison based on infection status or differential exposure intensity, the pandemic cannot be interpreted here as an independently tested exposure. It should instead be regarded as the broader contextual background in which the observed associations between sleep, stress, and pregnancy outcomes were measured.

### 4.5. Pandemic-Era Benchmarking of Sleep and Stress Burden

The COVID-19 period provides a clinically meaningful context for interpreting the magnitude of sleep and stress disturbances observed in our high-risk cohort. When benchmarked against pre-pandemic pregnancy data, our participants demonstrated a substantially higher sleep-burden profile. In a large meta-analysis of pregnancy studies using PSQI (pre-pandemic), the pooled average PSQI score was ~6.1 and the prevalence of poor sleep quality (PSQI ≥ 5) was ~45.7% [48].

Although the present study did not include internal pre-pandemic or post-pandemic comparison groups, external benchmarking against published pregnancy cohorts may help contextualize the magnitude of sleep and stress burden observed in our sample. These comparisons are descriptive only and should not be interpreted as direct causal evidence of a pandemic effect within the present cohort.

In contrast, our cohort had persistently elevated PSQI values (8.1–8.5) and a very high prevalence of poor sleep (89%), consistent with reports from pregnancy cohorts studied during the COVID-19 era where poor sleep was frequently reported and reached ~88% in some samples [49].

A similar pattern is observed for perceived stress. In a non-pandemic cohort assessed with PSS-10 at approximately mid–late pregnancy, mean stress values were ~11.4 [50]. During the COVID-19 outbreak, multinational data reported higher average perceived stress (PSS ≈ 14.1 ± 6.6) among pregnant women, while comparative Dutch cohort data showed markedly higher PSS-10 levels among women who explicitly attributed stress to the pandemic (mean ~15.6) compared with those who did not (~10.3) [51].

In our cohort, mean PSS-10 was even higher (16.2–17.0), suggesting a substantial psychosocial burden in this clinically high-risk population observed during the COVID-19 period.

Taken together, these descriptive comparisons suggest that the cohort was characterized by a high burden of sleep disturbance and perceived stress. However, because these data derive from external studies rather than an internal comparator design, they should be viewed as contextual benchmarks rather than evidence that the pandemic itself caused the observed associations.

All data highlighting pre-pandemic benchmarking and comparison is visualized in Table 7.

### 4.6. Limitations and Future Prospects

Several limitations must be considered when interpreting these findings.

First, the single-center design and moderate sample size limit generalizability. Although the study was adequately powered to detect medium effect sizes, larger multicenter cohorts are needed to confirm these associations across diverse populations. Another important limitation is the absence of pre-pandemic and post-pandemic comparator groups. For this reason, the COVID-19 period should be interpreted as the contextual background of recruitment rather than as a directly evaluated exposure.

Furthermore, while we included validated subjective and objective measures of sleep and stress, other potential confounders such as depressive symptoms, social support, and sleep-disordered breathing were not assessed. The absence of formal polysomnography and reliance on wearable-derived estimates may introduce measurement variability, although prior studies have shown acceptable concordance for total sleep time and efficiency.

Wearable sleep was collected over 72 h at each timepoint to reduce participant burden; longer monitoring windows (7–14 days) would better capture habitual sleep and weekday-to-weekend variability, and should be prioritized in future studies.

Cortisol profiling was based on three daily samples over two days; while this supports CAR and slope estimation, higher-resolution sampling would strengthen inference about HPA-axis dynamics.

Third, salivary cortisol was collected over two days, providing only a short-term snapshot of HPA axis activity. Repeated measurements throughout gestation could better capture chronic stress exposure.

Additionally, the pandemic-specific context may limit direct extrapolation to non-crisis settings; however, it also strengthens the study’s relevance by capturing maternal vulnerability during a period of sustained psychosocial stress.

Finally, the study’s focus on women already at moderate or high clinical risk limits its applicability to the general obstetric population. Nevertheless, this homogeneous high-risk cohort minimizes confounding from low-risk factors and underscores the value of behavioral markers within already-compromised pregnancies.

Future research should aim to validate these findings in larger, multicenter cohorts with diverse risk profiles. Incorporating more granular and objective assessments of sleep, such as actigraphy combined with polysomnography, and longitudinal monitoring of stress biomarkers throughout pregnancy may help clarify the dynamic interactions between sleep, stress, and pregnancy outcomes. Moreover, interventional studies exploring whether targeted sleep hygiene or stress reduction interventions in high-risk pregnancies can positively influence fetal growth and maternal outcomes represent an important area for future investigation.

## 5. Conclusions

This study demonstrates that combining sleep and stress parameters yields a more accurate prediction of preeclampsia and fetal growth restriction than analyzing either domain independently. The results highlight the interdependence between behavioral and neuroendocrine pathways in pregnancy adaptation and provide a rationale for incorporating sleep and stress assessment into comprehensive obstetric risk stratification.

Integrating wearable-based sleep metrics and salivary cortisol monitoring within antenatal programs could enable earlier identification of vulnerable pregnancies and inform targeted preventive strategies.

This study was conducted during the COVID-19 period, but the absence of pre-pandemic and post-pandemic comparator groups means that the pandemic should be interpreted as study context rather than as a directly tested explanatory factor. Within that context, integrated sleep and stress assessment improved prediction of preeclampsia and impaired fetal growth in high-risk pregnancies, supporting the clinical relevance of behavioral and psychophysiological monitoring in antenatal care.

## Figures and Tables

**Table 1:** Participant demographics.

Variable	Mean ± SD/n (%)
Age (years)	29.8 ± 4.5
BMI (kg/m^2^)	27.3 ± 3.2
Gestational age at inclusion (weeks)	16.2 ± 0.4
Nulliparity	88 (51.9%)
History of preeclampsia	28 (16.5%)
Active smoking	38 (22.4%)
Socioeconomic status	
Low	63 (37.1%)
Medium	82 (48.2%)
High	25 (14.7%)
Education level	
High school or below	110 (64.7%)
University degree	60 (35.3%)

Descriptive statistics are presented as mean (standard deviation) for continuous variables and number (percentage) for categorical variables.

**Table 2:** Preeclampsia characteristics in the study cohort.

Variable	n (%) or Mean (SD)
Total cases of preeclampsia	47 (27.8%)
Early-onset preeclampsia (<34 weeks)	9 (5.6%)
Late-onset preeclampsia (≥34 weeks)	38 (22.2%)
Severe preeclampsia cases	19 (11.1%)
Mean gestational age at diagnosis (weeks)	34.8 (2.1)
Mean birth weight in preeclampsia cases (g)	2400 (450)
NICU admission in preeclampsia cases (%)	28 (60%)

Descriptive statistics are presented as number (percentage) for categorical variables and mean (standard deviation) for continuous variables. Preeclampsia cases were classified according to the American College of Obstetricians and Gynecologists (ACOG) diagnostic criteria. No inferential statistical tests were applied within this table; data reflect observed frequencies and central tendencies in the cohort.

**Table 3:** Subjective and biological stress assessments.

Variable	Mean at 16–18 Weeks (SD)	Mean at 24–26 Weeks (SD)
PSS-10 score	16.2 (4.1)	17.0 (4.3)
GAD-7 score	6.8 (2.3)	7.1 (2.4)
Morning cortisol (nmol/L)	12.4 (3.5)	13.1 (3.8)
CRP (mg/L)	4.3 (1.2)	4.5 (1.3)

Mean values with standard deviation are shown. Comparisons over time were descriptive.

**Table 4:** Maternal sleep assessment data.

Variable	Mean at 16–18 Weeks (SD)	Mean at 24–26 Weeks (SD)
PSQI score	8.1 (2.1)	8.5 (2.3)
ESS score	7.5 (2.0)	7.8 (2.2)
Total sleep time (hours)	6.2 (0.8)	6.0 (0.9)
Sleep efficiency (%)	82.3 (5.2)	80.9 (5.5)
Nocturnal awakenings (n/night)	3.8 (1.1)	4.0 (1.2)

Mean values with standard deviation are shown. Comparisons over time were descriptive.

**Table 5:** Correlation of maternal sleep and stress parameters with outcomes.

Outcome	Predictor Variable	Correlation Coefficient (r)	*p*-Value	q-Value (FDR)
Birth Weight (g)	PSQI (score)	−0.34	0.008 *	0.096
	ESS (score)	−0.16	0.24	0.576
	PSS-10 (score)	0.20	0.149	0.467
	GAD-7 (score)	0.03	0.803	0.876
	Cortisol (nmol/L)	0.22	0.113	0.467
	CRP (mg/L)	−0.07	0.612	0.816
Gestational Age at Delivery (weeks)	PSQI (score)	−0.28	0.04 *	0.240
	ESS (score)	−0.25	0.03 *	0.240
	PSS-10 (score)	0.14	0.308	0.672
	GAD-7 (score)	0.01	0.949	0.949
	Cortisol (nmol/L)	0.19	0.158	0.467
	CRP (mg/L)	−0.05	0.713	0.834
NICU Admission (yes = 1)	PSQI (score)	0.06	0.673	0.834
	ESS (score)	0.05	0.730	0.834
	PSS-10 (score)	0.12	0.384	0.709
	GAD-7 (score)	0.08	0.556	0.803
	Cortisol (nmol/L)	0.28	0.002 *	0.048
	CRP (mg/L)	0.18	0.175	0.467
Preeclampsia development (yes = 1)	PSQI (score)	0.09	0.514	0.803
	ESS (score)	0.02	0.880	0.918
	PSS-10 (score)	0.11	0.432	0.741
	GAD-7 (score)	0.08	0.569	0.803
	Cortisol (nmol/L)	0.22	0.119	0.467
	CRP (mg/L)	0.13	0.356	0.709

Pearson correlation analysis between maternal sleep and stress parameters and pregnancy outcomes. The correlation coefficient (r) indicates the strength and direction of the linear relationship, with values near ±1.0 denoting strong correlations and values near 0 indicating weak associations. Statistically significant correlations (*p* < 0.05) are marked with an asterisk (*).

**Table 6:** Multivariate regression analysis of sleep and stress parameters on maternal and fetal outcomes.

Outcome	Model	R-Squared/Pseudo R-Squared	*p*-Value (Model)
Preeclampsia	Sleep only	0.039	0.279
	Stress only	0.048	0.536
	Sleep + Stress	0.150	0.015 *
Birth Weight (g)	Sleep only	0.130	0.028 *
	Stress only	0.119	0.177
	Sleep + Stress	0.260	0.003 *
Gestational Age at Delivery (weeks)	Sleep only	0.020	0.593
	Stress only	0.035	0.778
	Sleep + Stress	0.070	0.738
NICU Admission (yes = 1)	Sleep only	0.036	0.647
	Stress only	0.074	0.273
	Sleep + Stress	0.106	0.296

All significant *p*-Value (<0.05) was marked with (*).

**Table 7:** Pandemic-era benchmarking of sleep and stress burden in pregnancy: comparison of our high-risk cohort with pre-pandemic and COVID-19 cohorts.

Domain (Instrument)	Our Cohort (High-Risk)	Pre-Pandemic Pregnancy Benchmark	COVID-19 Era Pregnancy Benchmark
Sleep quality (PSQI), mean	8.1 (16–18 weeks); 8.5 (24–26 weeks)	~6.07 pooled mean PSQI in pregnancy [48]	Markedly elevated sleep disturbance; up to 88% poor sleep reported in COVID-19 pregnancy cohorts [49]
Poor sleep prevalence (PSQI ≥ 5)	89%	45.7% pooled prevalence (meta-analysis) [48]	88% poor sleep (PSQI > 5) during COVID-19 pregnancy [49]
Perceived stress (PSS-10), mean	16.2 (16–18 weeks); 17.0 (24–26 weeks)	11.38 mean PSS-10 at ~27 gestational weeks [48]	14.1 ± 6.6 in multinational COVID-19 [50] pregnancy cohort; 15.62 ± 6.44 when stress attributed to COVID-19 [51]
Anxiety (GAD-7), mean	6.8 (16–18 weeks); 7.1 (24–26 weeks)	Not consistently reported in pre-pandemic PSQI/PSS benchmarks	11% of pregnant women with GAD-7 ≥ 10 during COVID-19 [44,50]

Footnote: Values for our cohort are extracted from Table 3 and Table 4 of the present manuscript (assessed at 16–18 and 24–26 gestational weeks). PSQI poor sleep was defined as PSQI ≥ 5. PSS values are presented on the PSS-10 scale (0–40). External comparators were selected as widely cited pregnancy benchmarks and/or COVID-19-era pregnancy cohorts using the same instruments. Between-study differences in population risk profile, gestational timing, recruitment method (clinic vs. online), and country-level pandemic restrictions may contribute to heterogeneity; therefore, comparisons are intended to contextualize magnitude rather than provide causal attribution.

## Data Availability

The raw data supporting the conclusions and results of this article will be made available on request.

## Supplementary Materials

The following supporting information can be downloaded at: https://www.mdpi.com/article/10.3390/life16040605/s1, Table S1: Baseline comparison of retained vs non-retained participants; Table S2: Internal consistency (Cronbach’s alpha) and stability (ICC) of instruments; Table S3: Convergent validity: correlations between PSQI and Fitbit sleep metrics; Table S4. Wear-time compliance and weekday composition for wearable sleep (mock); Table S5. Within-person change models (Δ 24–26 minus 16–18 weeks) predicting outcomes (mock).; Table S6. Derived cortisol metrics (CAR, diurnal slope, AUCg) by outcome group and exploratory associations.

## Abbreviations

ACOG	American College of Obstetricians and Gynecologists
BMI	Body Mass Index
CRP	C-Reactive Protein
ESS	Epworth Sleepiness Scale
FITBIT	(Brand Name) Fitbit Sense 2 Sleep Tracker
GAD-7	Generalized Anxiety Disorder 7-item Scale
HPA	Hypothalamic–Pituitary–Adrenal (Axis)
NICU	Neonatal Intensive Care Unit
PSS-10	Perceived Stress Scale—10-item version
PSQI	Pittsburgh Sleep Quality Index
ROC	Receiver Operating Characteristic
SD	Standard Deviation
SDB	Sleep-Disordered Breathing
SPSS	Statistical Package for the Social Sciences
