# Supplementary material for: Sleep Disruption, Psychological Stress, and Preeclampsia in High-Risk Pregnancies During the COVID-19 Era
Source: Life (Basel). 2026 Apr 5;16(4):605. doi: 10.3390/life16040605 (PMC13117767; doi:10.3390/life16040605)
Supplement: Supplementary file 1 [file life-16-00605-s001.zip › Table_S1.pdf]

Table S1. Baseline comparison of retained vs non-retained participants

Variable	Retained (n=170)	Not retained (n=128)	p-value	SMD
Age, years (mean ± SD)	29.8 ± 4.5	30.4 ± 4.9	0.280	0.13
BMI, kg/m² (mean ± SD)	27.3 ± 3.2	27.9 ± 3.6	0.137	0.18
Nulliparity, n (%)	88 (51.9)	70 (54.7)	0.702	0.06
Active smoking, n (%)	38 (22.4)	34 (26.6)	0.482	0.10
History of preeclampsia, n (%)	28 (16.5)	19 (14.8)	0.825	0.04
Socioeconomic status, n (%)	-	-	0.084	—
Low	63 (37.1)	62 (48.4)	N/A	—
Medium	82 (48.2)	55 (43.0)	N/A	—
High	25 (14.7)	11 (8.6)	N/A	—
Low socioeconomic status (binary: low vs non-low), n (%)	63 (37.1)	62 (48.4)	0.048	0.23
Education level, n (%)	-	-	0.103	—
High school or below	110 (64.7)	95 (74.2)	N/A	—
University degree	60 (35.3)	33 (25.8)	N/A	—
High school or below (binary), n (%)	110 (64.7)	95 (74.2)	0.103	0.21

p-values from Welch t-test (continuous) and chi-square test (categorical). SMD = standardized mean difference (absolute values shown); SMD >0.10 suggests meaningful imbalance.
