# Supplementary material for: Sleep Disruption, Psychological Stress, and Preeclampsia in High-Risk Pregnancies During the COVID-19 Era
Source: Life (Basel). 2026 Apr 5;16(4):605. doi: 10.3390/life16040605 (PMC13117767; doi:10.3390/life16040605)
Supplement: Supplementary file 1 [file life-16-00605-s001.zip › Table_S2.pdf]

Table S2. Internal consistency (Cronbach's alpha) and stability (ICC) of instruments

Instrument	Cronbach's alpha at 16–18 weeks	ICC (16–18 vs 24–26 weeks)	95% CI
PSQI	0.79	0.64	0.54–0.72
ESS	0.81	0.61	0.50–0.70
PSS-10	0.84	0.59	0.48–0.68
GAD-7	0.89	0.66	0.56–0.74
