# Supplementary material for: Sleep Disruption, Psychological Stress, and Preeclampsia in High-Risk Pregnancies During the COVID-19 Era
Source: Life (Basel). 2026 Apr 5;16(4):605. doi: 10.3390/life16040605 (PMC13117767; doi:10.3390/life16040605)
Supplement: Supplementary file 1 [file life-16-00605-s001.zip › Table_S3.pdf]

Table S3. Convergent validity: correlations between PSQI and Fitbit sleep metrics

Time point	Pairs	r	p-value
16–18 weeks	PSQI vs Total sleep time (h)	−0.32	<0.001
16–18 weeks	PSQI vs Sleep efficiency (%)	−0.27	0.001
24–26 weeks	PSQI vs Total sleep time (h)	−0.28	0.001
24–26 weeks	PSQI vs Sleep efficiency (%)	−0.24	0.004
