# Supplementary material for: Sleep Disruption, Psychological Stress, and Preeclampsia in High-Risk Pregnancies During the COVID-19 Era
Source: Life (Basel). 2026 Apr 5;16(4):605. doi: 10.3390/life16040605 (PMC13117767; doi:10.3390/life16040605)
Supplement: Supplementary file 1 [file life-16-00605-s001.zip › Table_S4.pdf]

Table S4. Wear-time compliance and weekday composition for wearable sleep (mock).

Metric	16–18 weeks (n=170)	24–26 weeks (n=170)
Total wear time, hours (mean ± SD)	70.6 ± 3.2	69.8 ± 3.8
Valid nights (>=4 h), n (%)		
3 nights	163 (95.9)	159 (93.5)
2 nights	6 (3.5)	9 (5.3)
1 night	1 (0.6)	2 (1.2)
0 nights	0 (0.0)	0 (0.0)
Window includes at least one weekend night, n (%)	75 (44.1)	70 (41.2)

A valid night was defined as >=4 hours of wear during the sleep interval. Weekend night defined as Friday or Saturday night.
