# Supplementary material for: Sleep Disruption, Psychological Stress, and Preeclampsia in High-Risk Pregnancies During the COVID-19 Era
Source: Life (Basel). 2026 Apr 5;16(4):605. doi: 10.3390/life16040605 (PMC13117767; doi:10.3390/life16040605)
Supplement: Supplementary file 1 [file life-16-00605-s001.zip › Table_S5.pdf]

Table S5. Within-person change models (Δ 24–26 minus 16–18 weeks) predicting outcomes (mock).

Model	Predictor	Effect	95% CI	p-value
Preeclampsia (logistic)	ΔPSQI (per +1 point)	OR 1.22	1.05–1.42	0.009
Preeclampsia (logistic)	ΔPSS-10 (per +5 points)	OR 1.18	1.02–1.36	0.026
Preeclampsia (logistic)	ΔGAD-7 (per +2 points)	OR 1.10	0.96–1.26	0.162
Birth weight (linear)	ΔPSQI (per +1 point)	β −78 g	—	0.012
Birth weight (linear)	ΔPSS-10 (per +5 points)	β −52 g	—	0.044
Birth weight (linear)	ΔFitbit TST (per +1 hour)	β +41 g	—	0.118

Each model includes the baseline value of the same predictor at 16–18 weeks plus the Δ-change term. β shown for linear models; OR shown for logistic models.
