# Supplementary material for: Sleep Disruption, Psychological Stress, and Preeclampsia in High-Risk Pregnancies During the COVID-19 Era
Source: Life (Basel). 2026 Apr 5;16(4):605. doi: 10.3390/life16040605 (PMC13117767; doi:10.3390/life16040605)
Supplement: Supplementary file 1 [file life-16-00605-s001.zip › Table_S6.pdf]

Table S6. Derived cortisol metrics (CAR, diurnal slope, AUCg) by outcome group and exploratory associations

Metric	No preeclampsia (n=123)	Preeclampsia (n=47)	p-value
CAR (C30 − C0), nmol/L	4.6 ± 2.0	5.8 ± 2.1	0.004
Diurnal slope (C30 to bedtime), nmol/L/hour	−0.39 ± 0.14	−0.31 ± 0.12	0.011
AUCg (C0, C30, bedtime), nmol/L*hour	152 ± 41	168 ± 46	0.038

Exploratory models (adjusted for baseline cortisol value):

Outcome	Predictor	Effect	p-value
Preeclampsia	CAR (per +1 nmol/L)	OR 1.15	0.030
NICU admission	AUCg (per +10 units)	OR 1.09	0.041

CAR and slope averaged across two days at each timepoint; AUCg computed via trapezoidal rule across C0, C30, and bedtime samples.
